# Detection of volatile organic compounds associated with *E. coli* using headspace correlation gas chromatography

**DOI:** 10.1007/s00216-026-06382-9

**Published:** 2026-02-18

**Authors:** Wan Sin Heng, Maiken Ueland, Snehal R. Jadhav, Robert A. Shellie

**Affiliations:** 1 University of Tasmania, Tasmanian Institute of Agriculture, Launceston, TAS Australia; 2 University of Technology Sydney, Centre for Forensic Science, School of Mathematical and Physical Sciences, Ultimo, NSW Australia; 3 Deakin University, Deakin Centre for Advanced Food Science, School of Exercise and Nutrition Sciences, Burwood, VIC Australia; 4 ARC Training Centre for Hyphenated Analytical Separation Technologies (HyTECH), Hobart, TAS Australia

**Keywords:** Gas chromatography, *Escherichia coli*, GC × GC, Headspace, MVOC, Microbial volatile organic compound

## Abstract

A dynamic headspace correlation gas chromatography (HS-cGC) approach to detect volatile organic compounds associated with *Escherichia coli* (*E. coli*) in nutrient broth is described. *E. coli* identification was achieved by detecting microbial volatile organic compounds (mVOCs) produced by the bacteria in the sample matrix. Headspace of the test solution was introduced into the separation column up to 256 times in a single analysis using a pseudo-random binary modulation sequence (PRBS). A convoluted chromatogram was recorded and later cross-correlated with the injection pattern to generate a correlogram. Inoculated samples starting from single cell inoculum were distinguished from sterile controls after 11 h enrichment. Conventional culture-based plating methods need analysis time on the order of days, but HS-cGC reduced the time-to-detection by approximately one-full day compared to culture-based plating methods. Quantitative comparison of key figures of merit between dynamic HS-cGC and static headspace-comprehensive two-dimensional gas chromatography (HS-GC × GC) is also presented. Here mixed standards were analysed to determine linearity, method detection limit (MDL), limit of quantification (LOQ), and repeatability. The correlation technique demonstrated MDL in the range of 0.5 to 2.0 mg/L and LOQ 2.0 to 5.5 mg/L for the target compounds. These results surpassed the static HS-GC × GC which exhibited a higher MDL (20 to 40 mg/L) and LOQ (80 to 150 mg/L), with comparable linearity and repeatability. With analytical figures of merit 20–40 times better (lower MDL and LOQ) than the previously described GC × GC approach, the HS-cGC methodology is an attractive alternative for trace target analyte determination. HS-cGC has a high potential to complement conventional culture-based plating methods to ramp up surveillance to detect pathogens and/or spoilage microbes in the food setting.

**Graphical abstract:**

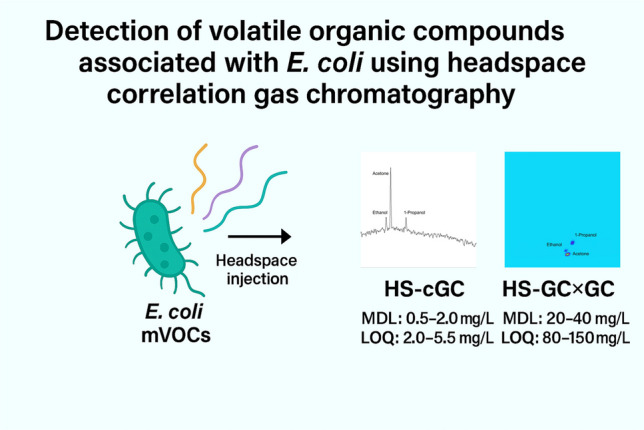

**Supplementary Information:**

The online version contains supplementary material available at 10.1007/s00216-026-06382-9.

## Introduction

The World Health Organization has challenged us to make food safety everyone's business [1]. Annually, it is estimated that approximately 600 million people will succumb to foodborne illness due to food contamination [1]. Early detection of pathogenic microorganisms in foods is a critical priority for reducing the incidence of foodborne illness. Traditionally, food testing laboratories have relied on culture-based methods to isolate and identify microorganisms. However, these methods are time-consuming and labour-intensive, which makes them challenging for time-sensitive analyses. *Escherichia coli* (*E. coli*) is one of the significant pathogens responsible for foodborne illness worldwide. Due to the high prevalence of foodborne illness outbreaks [2–6], this bacterium has become a primary target for food safety analyses. Microbial volatile organic compounds (mVOCs) are metabolites produced by bacteria, fungi, and moulds as a sign of active metabolism, and their presence could be a putative indicator of microbial growth. To date, more than 1000 organic compounds have been identified as mVOCs [7]. Amongst them, ethyl acetate, indole, 1-octanol, and 2-nonanone were associated with *E. coli* [8–11]. We recently described a multidimensional chromatography approach to rapidly detect *E. coli* in dairy milk by monitoring mVOCs [12]. Multidimensional chromatography was used to rapidly detect mVOCs associated with *E. coli* in dairy milk by monitoring acetaldehyde, ethanol, and 1-propanol using headspace-comprehensive two-dimensional gas chromatography (HS-GC × GC). Time-to-signal for HS-GC × GC was approx. 15.5 h (15 h of bacteria enrichment followed by 20 min HS equilibration and 8 min GC × GC analysis). Mean bacterial count (as tested) after 15 h enrichment was 8.0 ± 0.8 log CFU/mL. When HS analysis was started after 10 h of bacteria enrichment, the presence of bacteria was inconclusive despite the test solution containing 5.1 ± 0.5 log CFU/mL *E. coli* (as tested). It is postulated that the 10 h false-negative result was due to trace level mVOC production during the initial stages of bacteria growth. For instance, 1-propanol was below the method detection limit in all replicates at the 10 h time point. We postulate that a methodology with a method detection limit may improve earlier detection. The present investigation extends our previous experiment and explores opportunities to further reduce the time-to-signal without the use of time-consuming or complicated sample preparation.

Correlation gas chromatography (cGC) incorporates cross-correlation as a signal enhancement technique. Correlation GC was first conceptualised in 1967 for continuous gas analysis [13]. The continuous analysis aspect is highly advantageous because it enables dynamic headspace analysis without recourse to a sorbent trap. Unlike conventional chromatographic analysis where a single sample introduction (injection) takes place, many intra-analysis injections are made in correlation chromatography. Timing of each injection is determined by an input function known as pseudo-random binary sequence (PRBS), which consists of an array of “1 s” and “0 s”, whereby an injection takes place upon “1”, and no injection takes place upon “0”. The PRBS instructs a sampling device which modulates the sample flow at the inlet of the separation column. An arithmetically derived PRBS has a defined length and arrangement, but for all intents and purposes, the order of 1 s and 0 s appears to be random. After a defined period, the PRBS may be repeated to extend analysis time if necessary.

At first glance, the cGC detector output is incomprehensible due to the superimposed peaks from each injection. However, once cross-correlated with the known input sequence (i.e. demodulated with the PRBS), the result is a “correlogram” which resembles a conventional chromatogram. Cross-correlation is a signal averaging technique, under the assumption that the chromatogram of the test solution remains stationary (i.e. it does not change) but only delays in time. Cross-correlation (Eq. 1) involves determining the average product of one signal (i.e. the detector output) with the time-delayed version of another signal (i.e. the PRBS). The signal is sampled every δt with the new value being recalculated by averaging against the old data. The underlying assumption is that when a component is injected onto a separation column according to a binary sequence, the separation column will respond to each injection independently. This result in an identical output pulse to be observed after another delay [14].

1 $${R}_{xy}\left(\tau \right)=\frac{1}{T}\sum_{t=0}^{t=T}x\left(t-\tau \right)y\left(t\right)$$

where ${R}_{xy}\left(\tau \right)$ is the correlation coefficient, $y\left(t\right)$ is the detector output, *x* (*t*) is the input function (PRBS), $\tau$ is the delay, and $T$ is the number of time units.

Correlation chromatography effectively improves signal-to-noise ratio [15]: The detector response contains random noise that differs from the signal. The random component of the response cannot be cross-correlated, so during the procedure of calculating the correlogram, this noise will be averaged to zero.

With the capability to perform contiguous injections, cGC has found application in process analytical chemistry for rapid monitoring of changes in sample streams [16–18]. With the improved signal-to-noise ratio, cGC has also demonstrated utility for analysing components at trace levels [19–22]. Although cGC has proven effective in prior application, it has received limited attention from practitioners. This may be ascribed to unfamiliarity towards the approach and the need to apply the mathematical cross-correlation. However, advances and simplification of computer programming for instrument control, modern mathematical calculation environments, advanced pneumatic systems, developments in chromatographic modulation stemming from the development of GC × GC, etc. all make cGC highly accessible today. An example of the workflow employed in the present investigation for trace analysis is presented in Electronic Supplementary Materials S1. Detailed accounts of the principles and theory of correlation chromatography are described elsewhere [14, 23, 24].

## Materials and methods

All GC measurements were performed with an Agilent 7890B GC (Agilent, Mulgrave, Australia) and data were compared using Microsoft 365 Excel for all calculations. The instrument parameters were controlled by MassHunter software (Agilent). Conditions for the GC × GC were identical to those described in [12]. Quasi-stop-flow modulation was performed throughout. All cGC separations were performed on a 30 m × 0.25 mm I.D. (1.4 μm film thickness) BPX-Volatiles capillary column (Trajan Scientific and Medical, Ringwood, Australia). Hydrogen was used as an inert carrier gas with a split ratio of 100:1 and a flow rate of 2.5 mL/min. The GC oven was maintained isothermally at 50 °C. The detector temperature was held at 250 °C, with FID gases set to 40 mL/min hydrogen, 450 mL/min air, and 10 mL/min nitrogen as make-up. The headspace extraction (gas-tight syringe) was performed at room temperature.

A laboratory-built setup specifically for this experiment was constructed. The headspace above the test solution was sampled at room temperature directly from a 13 mL glass reservoir by passing GC carrier gas through the sealed reservoir (see Electronic Supplementary Materials S2). The sample reservoir was manually exchanged between analyses. The experimental setup was constructed using a 3-way solenoid valve (Agilent, G2399-60610), an Arduino Uno R3 as the controller (Arduino, Italy), and a sample chamber. The sample introduction device was directly mounted to the split/splitless injector with a heated sample port of 250 °C. Headspace was sampled and injections controlled using the 3-way valve with the predetermined PRBS timing parameters controlled by the Arduino board connected to a personal computer via USB. Specific timing parameters are described in the “Results and discussion” section.

A timing control program was written in the Arduino Integrated Development Environment (IDE). Injection sequences were constructed according to the basis of a virtual linear feedback shift register. The 9-bit pseudo-random sequence consists of 512 elements, with 50% (256) of the elements being 0 and the other 50% (256) of the elements being 1. Injections of samples were only performed when the elements were equal to 1. The time-to-inject (δt) was set to 3 s. The sequence was written in MATLAB Version: 9.14.0 (R2023a; see Electronic Supplementary Materials S3). The FID response was collected at 5 Hz using MassHunter. Demodulation was performed using Microsoft Excel (Microsoft, Washington, USA). Demodulated data were then processed using OriginPro 2024b (OriginLab Corporation, Massachusetts, USA) for correlogram presentation and peak integration.

Acetone (LC1003-G4L 99.9%) was purchased from ChemSupply (Gilman, Australia). Ethanol (AJA 214-2.5PL 99.5%) was purchased from Thermo Fisher Scientific (Scoresby, Australia). 1-Propanol (402893 99.5%) was purchased from Sigma-Aldrich (Macquarie Park, Australia). An aqueous 10,000 mg/L stock solution of the three standards was prepared and appropriately diluted in Milli-Q water to make calibration standards. Calibration solutions were prepared as follows: 1, 10, 20, 30, and 50 mg/L for cGC and 1, 10, 50, 100, and 500 mg/L for GC × GC. Each calibration solution was analysed in triplicate. MDL, LOQ, and repeatability (peak area relative standard deviation) were determined by using replicated spiked test solutions (*n* = 9) according to the EPA procedure [25]. Spiking concentrations used were as follows: for ethanol, 5 mg/L for cGC and 90 mg/L for GC × GC; for acetone, 2 mg/L for cGC and 75 mg/L for GC × GC; for 1-propanol, 5 mg/L for cGC and 90 mg/L for GC × GC.

Laboratory strain *E. coli* ATCC 25922 was used for the practical application study. Bacteria were recovered from freezer stock by aseptically streaking onto nutrient agar (CM0003B, Thermo Fisher Scientific) and incubated overnight at 37 °C. For each experiment, a fresh 24 h culture was revived on a nutrient agar plate and adjusted to 0.5 McFarland Standard (equivalent to 1.5 × 10^8^ CFU/mL) in sterile saline solution (0.85%), measured with a DensiCheck instrument (bioMérieux, North Ryde, Australia).

Nutrient broth (CM0001B, Thermo Fisher Scientific) was used as the sample matrix here and prepared according to the manufacturer’s guidelines and sterilised by autoclaving at 121 °C for 15 min. Sample solutions were prepared by diluting standardised *E. coli* solutions in sterile saline to achieve the desired inoculation concentration of 1 CFU/mL in the sample matrix. The sample solution was then incubated at 37 °C and was sampled across four timepoints of 5, 8, 11, and 14 h. At each timepoint, 5 mL of the inoculated sample solution was transferred to a 13 mL glass reservoir for the analysis and replicated accordingly (*n* = 3). Drop plate method [26] was used to determine the number of viable bacteria present in each test solution (see Electronic Supplementary Materials S4). Eosin methylene blue (EMB) agar was used as the selective and differential media for isolating the *E. coli* in the spiked nutrient broth. To calculate the CFU/mL, results for the replicates (*n* = 3). were averaged. For all method development experiments, reference stock solutions were utilised, whereas the bacterial samples were exclusively used in the timepoint study.

## Results and discussion

Initial method development of the HS-cGC technique involved design and testing of a sampling device to hold the test solution and allow for intermittent transfer of the headspace above the solution into the chromatograph (see Electronic Supplementary Materials S2). The sampling device was equipped with a 3-way solenoid valve, regulated by an Arduino microcontroller. In standby position (no injection; valve normally closed position), the carrier gas bypassed the glass sample chamber. Carrier gas was directed through the glass sample chamber by actuating the valve (injection; valve energised; normally open position) (see Electronic Supplementary Materials S5). This initiated a positive purge of the sample chamber, and an aliquot of headspace vapour was introduced to the chromatograph.

**Fig. 1:**
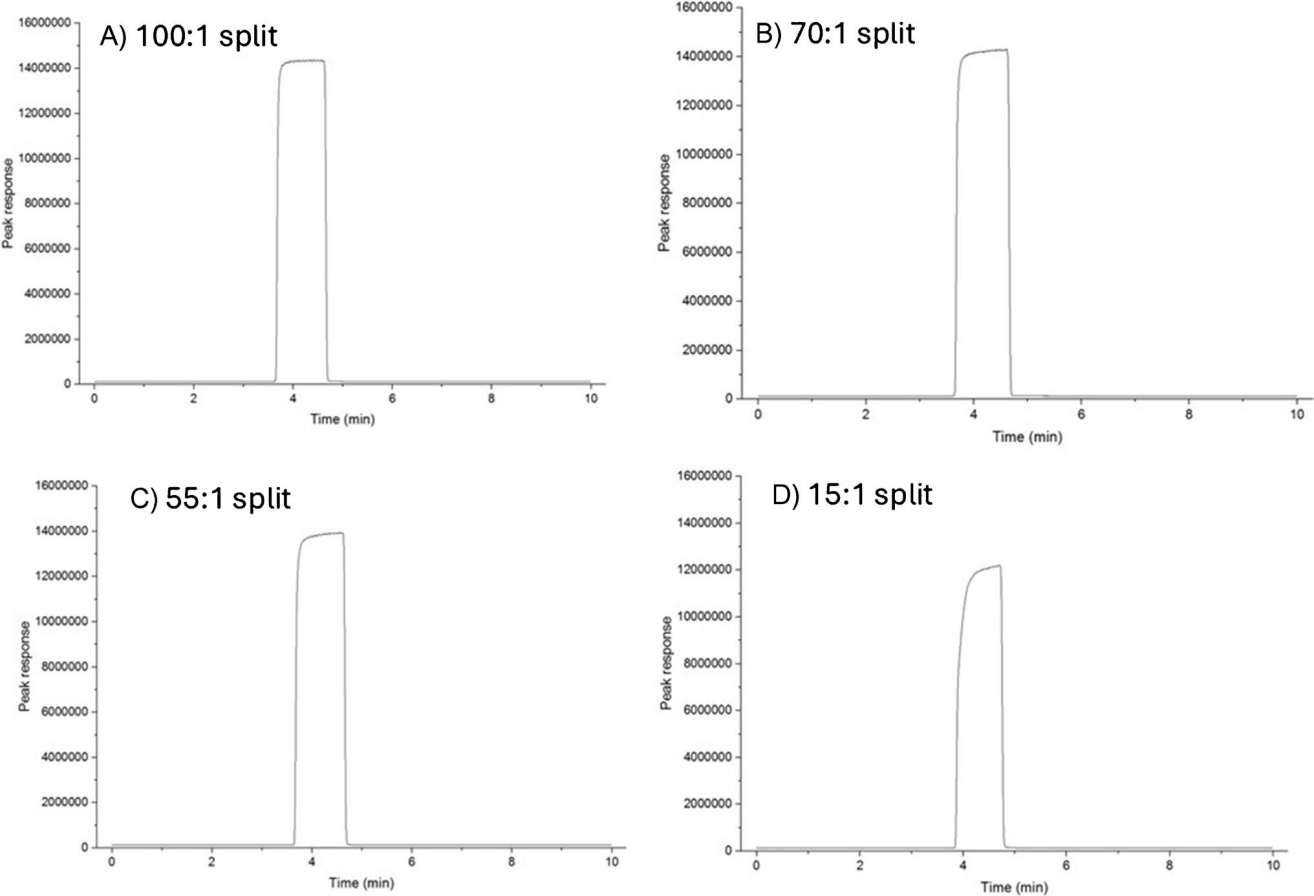
HS-GC chromatograms of 10,000 mg/L acetone in water using the sample introduction device with different applied split ratios

The valve switching protocol was first examined to verify correct operation and optimal sample delivery. This is one of the major considerations for a successful correlation application since poor valve operation can lead to inconsistent sample delivery and introduce injection errors into the system [27]. Column flow rate was set to 2.5 mL/min with a corresponding split ratio of 100:1. While a high split ratio may seem counterintuitive for trace analysis, the absolute size of the sample delivered to the column in a fixed time interval is independent of the split ratio. The split ratio increases monotonically with an increase in split vent flow (and therefore total flow); the column flow rate, which is determined as a function of head pressure, remains unchanged. Using a lower split flow may lose less sample to the split vent, but it will deliver the same volume on column. A high flow in the extra-column connecting tubing is necessary to deliver the sample pulse into the GC within the modulation time window. By using a high split ratio, higher total flow up to the separation column inlet was achieved, minimising deviation in the final chromatogram output. This was also highlighted by Trapp, where a split ratio as high as 200:1 was used to minimise the irregularities in the amount of sample injected [28]. This is demonstrated by the observation presented in Fig. 1, whereby the effect of the different split ratios is reflected in the peak shape of the injection. The peak in the chromatogram exhibits slight asymmetry in the lower split ratios, likely due to the reduced force to push the sample into the column. Higher total flow resulted in sharper leading and trailing edges of injection pulses, and the injection rapidly reached a steady-state response.

**Fig. 2:**
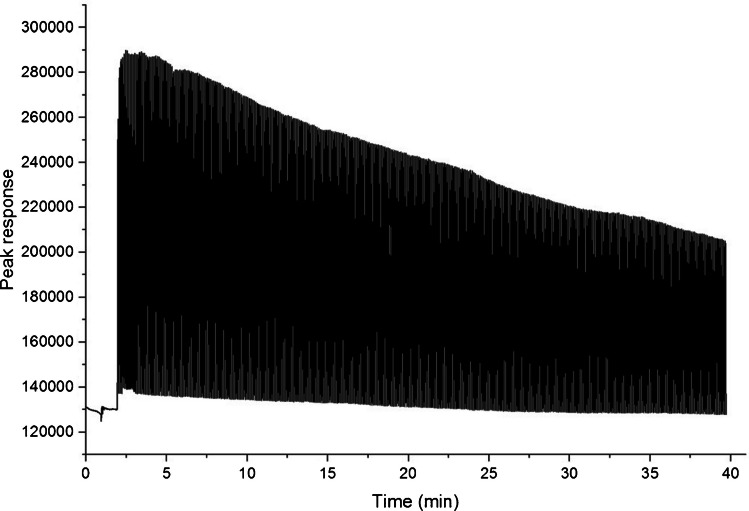
Contiguous 3 s injections to determine sample was sufficient to not be exhausted in a typical 40 min analysis

It is important to employ a modulation sequence that covers the last eluted peak to ensure accurate construction of the correlogram, while providing sufficient data points to effectively average out the random noise. The time interval (δt) must be determined beforehand, since this will also dictate the duration of GC runtime. Timing intervals between 600 ms and 60 s were investigated (see Electronic Supplementary Materials S6) where bandwidth increased monotonically with increasing modulation time. While the longer modulation time allows for greater sample introduction into the system, it also raises concern regarding reduced peak resolution, and sample depletion (i.e. sample can be exhausted before the modulation sequence finishes). Conversely, shortening the modulation time requires more frequent and rapid valve switching, which may compromise the longevity of the valve and increase the risk of injection error. Shorter injection intervals reduce the overall experimental analysis time but may lead to loss of chromatographic structure in the later part of the chromatogram due to peak overlap [28]. Too rapid modulation may also allow sample to pass through unmodulated giving a poor result since the length of the valve actuation is less than the length of the code event [23]. Risk of sample depletion at a higher total flow rate was assessed by performing injections using a regular binary sequence for 40 min (valve on/off every 3 s) and confirmed there was sufficient sample in the sampling device to avoid exhausting the test solution during analysis (Fig. 2).

**Fig. 3:**
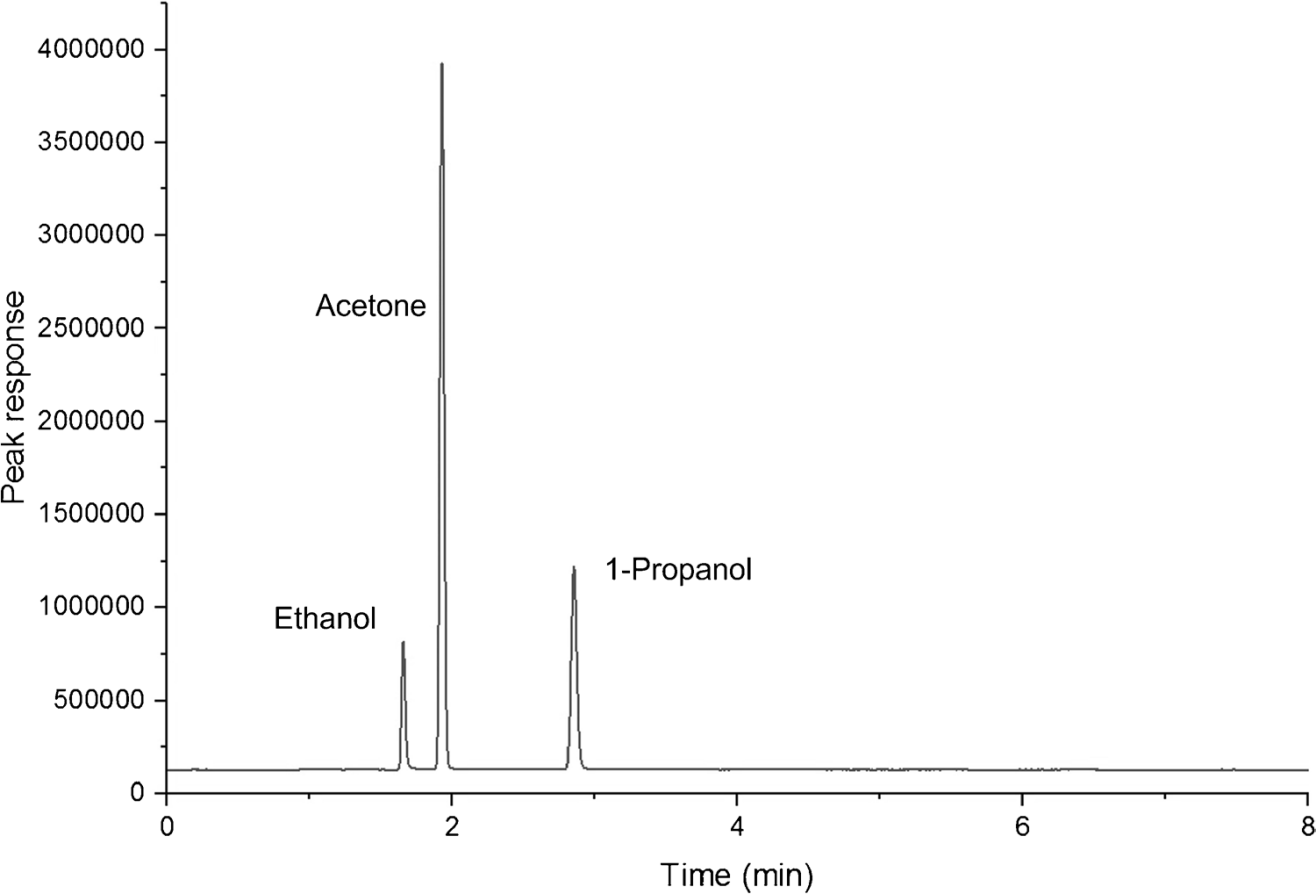
One-dimensional chromatogram of the test mixture. Ethanol, *t*_*R*_ = 1.7 min; acetone, *t*_*R*_ = 1.9 min; 1-propanol, *t*_*R*_ = 2.9 min

Separation column and stationary phase selection was informed by using the Pro EZGC Chromatogram Modeler software (Restek Corporation, USA), with the parameters of the column length, dimension, and film thickness tailored to maximise separation of target analytes. Adequate separation was confirmed using reference standards (acetone, ethanol, and 1-propanol) using a BPX-Volatiles GC column (Fig. 3).

After confirming the column’s separation capability, an initial attempt to perform cGC was made using narrow injections (*t* = 100 ms) and (δ*t* = 5 s), but the resulting correlogram exhibited significant baseline noise (see Electronic Supplementary Materials S7). This was later determined to be the result of “incomplete” modulation [29]. The initial approach was mistakenly undertaken due to the lack of clarity in the literature surrounding the concept of modulation and demodulation. In cGC, block-shaped peak injections are essential for successful demodulation [14, 23, 30]. For instance, if the sequence of events was 1, 0, 0, 1, 1, and δ*t* = 3 s, the valve must first open for 3 s, close for 6 s and open for 6 s, rather than open and close within 100 ms during the 3 s period. Modulation and demodulation were effectively implemented using a corrected 9-bit modulation sequence of 511 elements and 256 injections, with injection intervals δt = 3 s and a final run time of 35.5 min (see Electronic Supplementary Materials S7). This is consistent with optimised conditions described elsewhere [28].

**Fig. 4:**
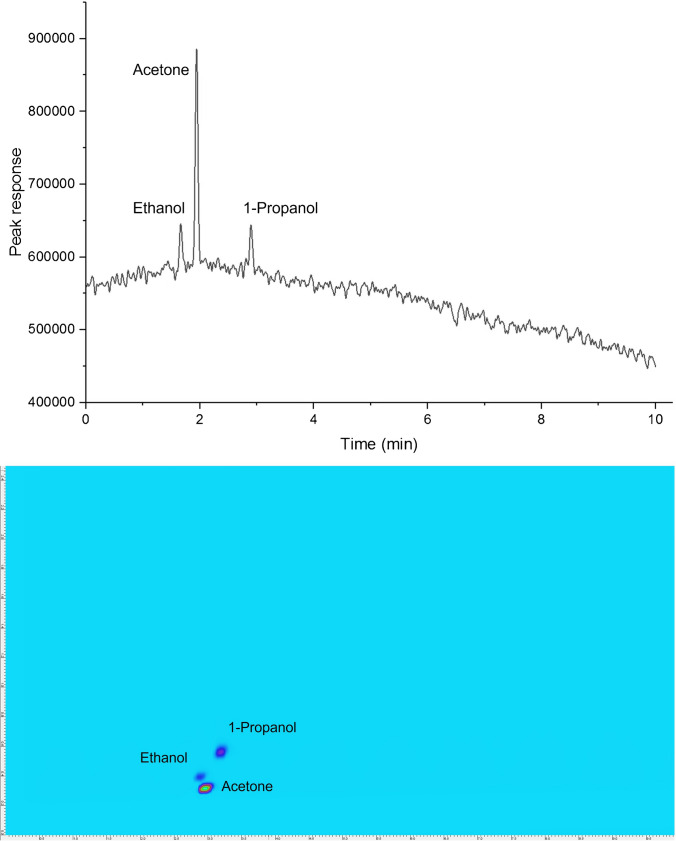
Typical chromatograms for mixed-standard test solutions for cGC (top) and GC × GC (bottom). Colour scale for GC × GC set at Min: 25.24 and Max: 956.43

Upon establishing the correlation approach, method performance of HS-cGC was carried out by evaluating key analytical figures of merit and compared with HS-GC × GC across all figures to benchmark performance (Table 1). An example correlogram for the two techniques can be seen in Fig. 4. It is important to note variation in the headspace sampling procedure between approaches. The HS-GC × GC technique incorporated static headspace extraction where an aliquot of the equilibrated headspace was transferred to the GC using a gas-tight syringe. On the other hand, HS-cGC represents room temperature dynamic headspace extraction and multiple injections of the headspace of the sample were injected within a single run without a pre-defined equilibration step between subsequent injections. Linearity was studied by analysing a series of mixed-standard test solutions of five different concentration levels, with each level tested in triplicate. The volatile standards analysed by HS-cGC and HS-GC × GC both showed highly satisfactory linearity (R^2^ ≥ 0.994 and R^2^ ≥ 0.999 respectively) across a concentration range spanning 1 to 500 mg/L. Mean recovery was estimated by spiking nine test replicates according to the EPA method. Although separate method blanks were not analysed, Milli-Q water was used as the clean matrix for all spiked sample preparations. Highly satisfactory spike recoveries were obtained ranging from 80 to 120% for HS-GC × GC and 70 to 95% for HS-cGC. Acetone had the lowest recoveries amongst the three compounds. MDL and LOQ were also evaluated, with MDL defined as the minimum measured concentration of a substance that can be reported with 99% confidence above background levels [25]. In line with typical conventions, LOQ represents the minimum amount of the analyte which can be quantitatively determined with an acceptable level of precision and accuracy [31]. LOQ was mathematically defined as equal to 10 times the standard deviation of the results of the spiked matrix. For GC × GC, the method produced MDL of 20 to 40 mg/L and LOQ of 80 to 150 mg/L. For cGC, the method delivered approximately one order of magnitude improved MDL (0.6 to 1.5 mg/L) and LOQ (2.0 to 5.3 mg/L). The spike recoveries demonstrated for HS-cGC were all in the acceptable range (between 70 and 91%), however, slightly lower than the HS-GC × GC technique (86 to 120%). This discrepancy might be due to the different concentrations (GC × GC 75 mg/L spike) used in the spiking procedure, while a much lower spike level for the correlation technique (2 mg/L spike). Nevertheless, our motivation to evaluate HS-cGC was validated by the lower MDL (approximately 30-fold) and LOQ (approximately 26- to 37-fold) demonstrated in this application. HS-cGC had remarkably low detection and quantification limits compared to HS-GC × GC. These exceptional detection and quantification limits were achieved in the headspace environment which has a lower analyte concentration compared to the liquid phase before the migration to the headspace begins [32]. To be able to attain such high-quality outcomes demonstrates the excellence of the cGC approach.

**Table 1:** Key figures of merit for target volatiles using HS-cGC and comparison with HS-GC × GC

**Analyte**	**R**^**2**^ **(%)**	**MDL** **(mg/L)**	**LOQ** **(mg/L)**	**Recoveries** **(%)**	**Repeatability** **(% RSD)**^**#**^
**GC × GC**					
Acetone	0.998	21.63	74.68	86	11
Ethanol	0.999	42.48	146.67	117	14
1-Propanol	0.999	38.96	134.50	120	12
**cGC**					
Acetone	0.998	0.58	2.00	70	14
Ethanol	0.994	1.48	5.12	91	11
1-Propanol	0.994	1.52	5.25	91	12

^#^Repeatability of detected concentration for replicated spiked solutions (*n* = 9)

**Fig. 5:**
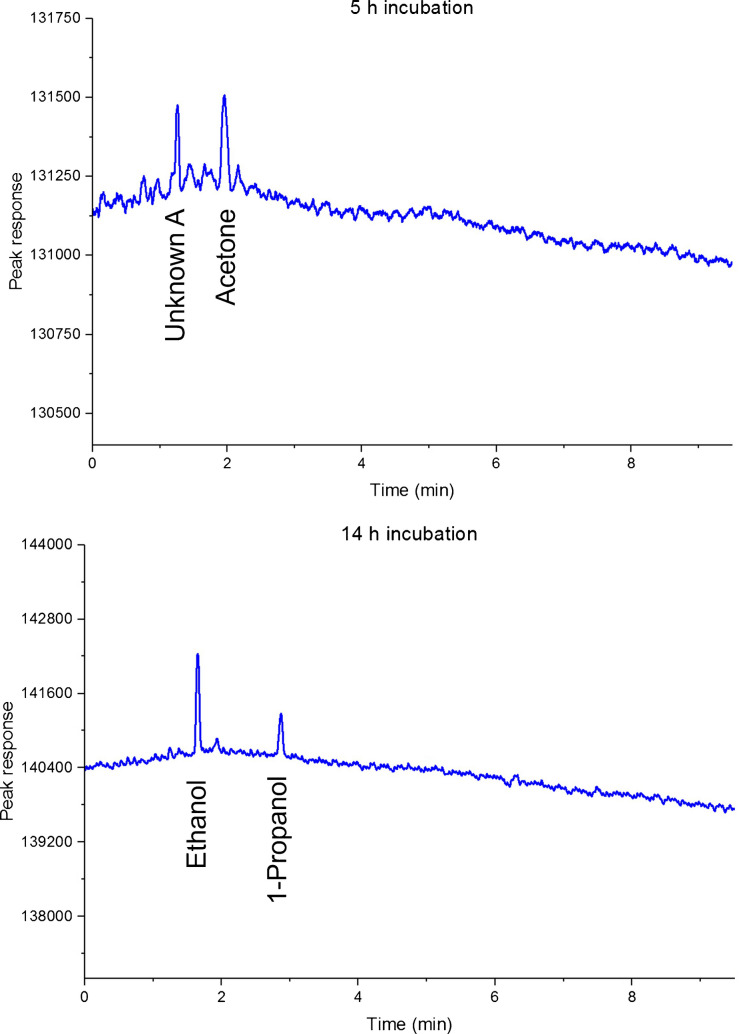
Correlogram comparing the control and the inoculated samples at 5 h and 14 h. Top result represents control samples while the bottom represents *E. coli* samples. The corresponding chromatograms for all time points are provided in Electronic Supplementary Materials SM 8

Figure 5 compares correlograms inoculated *E. coli* samples at two time points. Control samples (sterile nutrient broth without *E. coli* inoculation) and additional time points are presented in Electronic Supplementary Material SM 8. Authentic chemical references were used to verify the retention times of the target analytes. For the initial two time points, there were no differences detected between the controls and the *E. coli* samples, with only Unknown A and acetone being detected. After 11 h, and corresponding bacteria count of 7.8 ± 0.9 log CFU/mL, discrimination between the two sample groups was possible, with ethanol and 1-propanol present above MDL. After 14 h (8.9 ± 0.4 log CFU/mL), ethanol and 1-propanol were the predominant detected mVOCs. To ensure the reliability in the *E. coli* detection, only VOCs that appear in all replicates for a given condition were included in the final analysis. As in the previous study, the *E. coli* strain used in this study is a standard reference non-pathogenic strain, commonly employed for routine testing. In the present investigation, VOC analysis was conducted to compare treatments (control vs. spiked) within the same strain and culture conditions. Therefore, while inter-study VOC profiles may differ slightly due to strain level differences, the conclusions drawn in this study are based on internal comparisons of control versus spiked samples.

The HS-cGC approach was able to indicate bacteria presence as quickly as 11 h offering a time efficiency improvement compared to HS-GC × GC and a 77% time saving compared to traditional culture-based methodology (assuming 48 h plate and wait). Time-to-signal was reduced by 27% using HS-cGC (11 h) compared to HS-GC × GC (15 h). At these time points, a t-test showed no statistically significant difference in bacteria count (7.8 ± 0.9 vs 8.0 ± 0.8, p = 0.4). We propose that accumulation of mVOCs is time dependent as well as related to bacteria abundance. Evidence of mVOCs associated with *E. coli* was missed by HS-GC × GC until 15 h enrichment but apparent sooner using HS-cGCowing to lower MDL. It is noteworthy that all analyses commenced with a low concentration inoculum. HS-cGC poses a strong fit for the development of near real-time detection of samples with higher contamination, or where mVOC accumulation time has been longer. In this time where there is constant pressure for a shorter analysis time to achieve on-site analysis, the lower detection limit of the correlation approach presents a compelling feature in meeting these goals. When developing a technique, the total time involved in analysing the sample must be considered. This would involve the time required for sample preparation, sample introduction, separation, detection, data analysis, and reporting. From a food safety testing perspective, the main challenge to realising on-site analysis is the long enrichment period in the traditional culturing methodology. Despite having a faster GC analysis time, the enrichment process still lengthens the overall time-to-detection. By having an approach with a lower detection limit, this can significantly reduce the time required in the enrichment procedure that is aimed at increasing the amount of target bacterium in the food sample. Here, the term “trace level” refers to a concentration near the MDL or LOQ that were determined in the spiked test solutions. While the concentration of the target VOCs in the bacterial culture was not directly assessed, the MDL and LOQ studies provide a reliable estimate of the method’s ability to detect and quantify these compounds at low concentrations. With the cGC technique able to detect the target analytes at a trace level, it gives high confidence that the proposed approach will be able to identify the contamination at an early stage.

## Conclusion

A promising approach for detecting mVOCs was demonstrated. In this study, mVOCs associated with *E. coli* were successfully determined after an enrichment time of 11 h. The time needed for the HS-cGC analysis (36 min) is approximately equivalent to HS-GC × GC (28 min), but HS-cGC offers at least a magnitude lower MDL and LOQ than HS-GC × GC. Further work is required beyond this proof-of-principle study to determine method specificity for selected single bacterial agents. Notwithstanding, the described approach functions as a non-laborious detection approach that is capable of distinguishing between clean and contaminated samples. Overall, the development of such a system opens opportunities to create a reliable and (trans)-portable platform for performing at-sample detection. Furthermore, as a headspace methodology, the described approach could potentially be applied to other food types, including solid foods, aiding in monitoring microbial presence. Our proposed approach is not intended to replace conventional testing, rather to complement the existing workflows. The ability to rapidly assure clean samples will provide confidence to bypass the current time-consuming food testing step. Food safety analysts’ time will be freed from mundane repetitive analysis of true-negative samples, and free scientists to complete more complex and thought-intensive tasks. For potentially contaminated products alerted by HS-cGC, additional confirmation steps can be taken to verify the microbial identity.

The overarching aim of this study was to evaluate HS-cGC for rapid discrimination between clean and contaminated samples. To this end, the experiment was conducted using *E. coli* as a model organism and nutrient broth as an initial matrix. However, real-world applications often involve food products with complex compositions. These products may contain diverse components that contribute to a variety of background matrix and native microbial flora, which will interfere with the detection. As a result, re-evaluation will be required to establish a baseline specific to each food matrix. Nevertheless, the current outcomes show possible improvements in the efficiency in food safety evaluation.

## Supplementary Information

Below is the link to the electronic supplementary material.

Supplementary file1 (PDF 1.22 MB)

## Data Availability

The datasets generated and analysed during the current study are not publicly available but are available from the corresponding author on reasonable request.
