# Supplementary material for: Detection of volatile organic compounds associated with E. coli using headspace correlation gas chromatography
Source: Anal Bioanal Chem. 2026 Feb 18;418(10):3075–85. doi: 10.1007/s00216-026-06382-9 (PMC13144197; doi:10.1007/s00216-026-06382-9)
Supplement: Supplementary file 1 — Supplementary file1 (PDF 1.22 MB) [file 216_2026_6382_MOESM1_ESM.pdf]

Detection of volatile organic compounds associated with E. coli using headspace correlation gas chromatography

Wan Sin Heng [ad], Maiken Ueland [b], Snehal Jadhav [cd], and Robert A Shellie [*acd]

[a] University of Tasmania, Tasmanian Institute of Agriculture, Launceston, Tasmania, Australia

[b] University of Technology Sydney, Centre for Forensic Science, School of Mathematical and Physical Sciences, Ultimo, New South Wales, Australia

[c] Deakin University, Deakin Centre for Advanced Food Science, School of Exercise and Nutrition Sciences, Burwood, Victoria, Australia

[d] ARC Training Centre for Hyphenated Analytical Separation Technologies (HyTECH)

Electronic Supplementary Materials

*address for correspondence

Email: robert.shellie@utas.edu.au

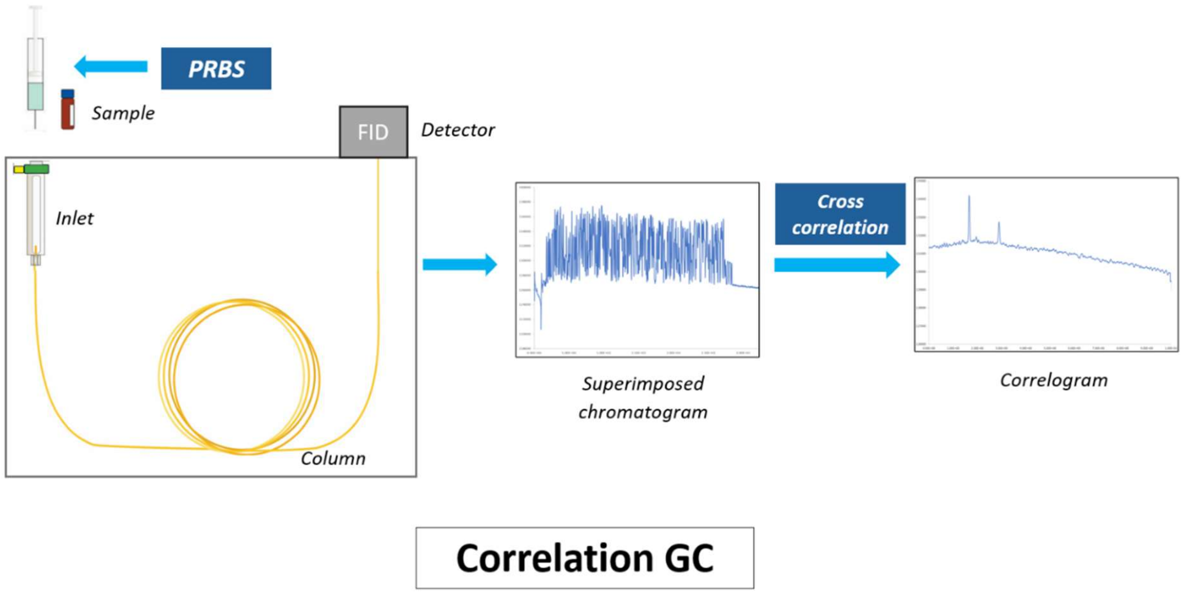

SM1 workflow for headspace-correlation gas chromatography employed in this study

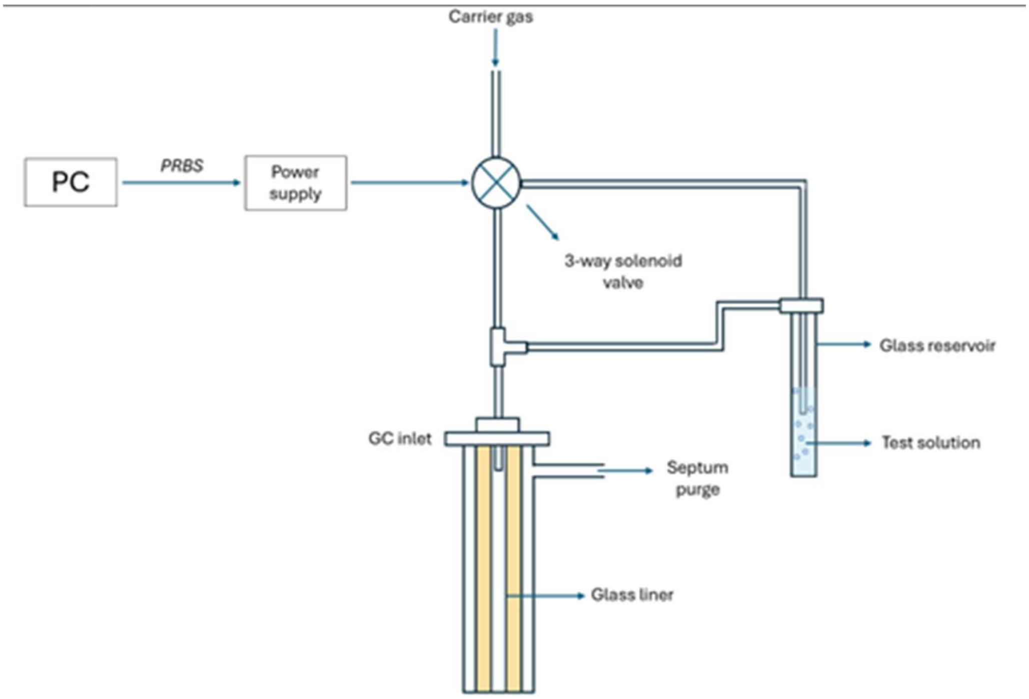

SM2 Schematic of the HS-cGC setup. The carrier gas line was directly immersed into the test solution to facilitate analyte transfer into the system.

```
const uint8_t prbs_sequence[] = {
    0, 1, 1, 0, 0, 0, 1, 0, 1,
    0, 1, 0, 0, 1, 0, 0, 0, 1,
    1, 1, 0, 0, 0, 1, 1, 0, 1,
    1, 0, 1, 0, 1, 0, 1, 1, 1,
    0, 0, 0, 1, 0, 0, 1, 1, 0,
    0, 0, 1, 0, 0, 0, 1, 0, 0,
    0, 0, 0, 0, 0, 0, 1, 0, 0,
    0, 0, 1, 0, 0, 0, 1, 1, 0,
    0, 0, 0, 1, 0, 0, 1, 1, 1,
    0, 0, 1, 0, 1, 0, 1, 0, 1,
    1, 0, 0, 0, 0, 1, 1, 0, 1,
    1, 1, 1, 0, 1, 0, 0, 1, 1,
    0, 1, 1, 1, 0, 0, 1, 0, 0,
    0, 1, 0, 1, 0, 0, 0, 0, 1,
    0, 1, 0, 1, 1, 0, 1, 0, 0,
    1, 1, 1, 1, 1, 1, 0, 1, 1,
    0, 0, 1, 0, 0, 1, 0, 0, 1,
    0, 1, 1, 0, 1, 1, 1, 1, 1,
    1, 0, 0, 1, 0, 0, 1, 1, 0,
    1, 0, 1, 0, 0, 1, 1, 0, 0,
    1, 1, 0, 0, 0, 0, 0, 0, 0,
    1, 1, 0, 0, 0, 1, 1, 0, 0,
    1, 0, 1, 0, 0, 0, 1, 1, 0,
    1, 0, 0, 1, 0, 1, 1, 1, 1,
    1, 1, 1, 0, 1, 0, 0, 0, 1,
    0, 1, 1, 0, 0, 0, 1, 1, 1,
    0, 1, 0, 1, 1, 0, 0, 1, 0,
    1, 1, 0, 0, 1, 1, 1, 1, 0,
    0, 0, 1, 1, 1, 1, 1, 0, 1,
    1, 1, 0, 1, 0, 0, 0, 0, 0,
    1, 1, 0, 1, 0, 1, 1, 0, 1,
    1, 0, 1, 1, 1, 0, 1, 1, 0,
    0, 0, 0, 0, 1, 0, 1, 1, 0,
    1, 0, 1, 1, 1, 1, 1, 0, 1,
    0, 1, 0, 1, 0, 1, 0, 0, 0,
    0, 0, 0, 1, 0, 1, 0, 0, 1,
    0, 1, 0, 1, 1, 1, 1, 0, 0,
    1, 0, 1, 1, 1, 0, 1, 1, 1,
    0, 0, 0, 0, 0, 0, 1, 1, 1,
    0, 0, 1, 1, 1, 0, 1, 0, 0,
    1, 0, 0, 1, 1, 1, 1, 0, 1,
    0, 1, 1, 1, 0, 1, 0, 1, 0,
    0, 0, 1, 0, 0, 1, 0, 0, 0,
    0, 1, 1, 0, 0, 1, 1, 1, 0,
    0, 0, 0, 1, 0, 1, 1, 1, 1,
    0, 1, 1, 0, 1, 1, 0, 0, 1,
    1, 0, 1, 0, 0, 0, 0, 1, 1,
    1, 0, 1, 1, 1, 1, 0, 0, 0,
    0, 1, 1, 1, 1, 1, 1, 1, 1,
    1, 0, 0, 0, 0, 0, 1, 1, 1,
    1, 0, 1, 1, 1, 1, 1, 0, 0,
    0, 1, 0, 1, 1, 1, 0, 0, 1,
    1, 0, 0, 1, 0, 0, 0, 0, 0,
    1, 0, 0, 1, 0, 1, 0, 0, 1,
    1, 1, 0, 1, 1, 0, 1, 0, 0,
```

```
     0, 1, 1, 1, 1, 0, 0, 1, 1,
     1, 1, 1, 0, 0, 1, 1
};

const int relayPin = 2;
const int buttonPin = 7;
const int statusLEDPin = 10;

bool sequenceRunning = false;
int numIterations = sizeof(prbs_sequence) / sizeof(prbs_sequence[0]);
int iterationCount = 0;

void setup() {
  pinMode(relayPin, OUTPUT);
  pinMode(buttonPin, INPUT_PULLUP);
  pinMode(statusLEDPin, OUTPUT);

  Serial.begin(9600);
}

void loop() {
  if (digitalRead(buttonPin) == LOW) {
    if (!sequenceRunning) {
      sequenceRunning = true;
      digitalWrite(statusLEDPin, LOW);
    } else {
      sequenceRunning = false;
      iterationCount = 0;
      digitalWrite(relayPin, LOW);
      digitalWrite(statusLEDPin, LOW);
      delay(3000);
    }
  }

  if (sequenceRunning) {
    unsigned long timestamp = millis();

    if (prbs_sequence[iterationCount] == 1) {
      digitalWrite(relayPin, HIGH);
      digitalWrite(statusLEDPin, HIGH);
      delay(3000);
    } else {
      digitalWrite(relayPin, LOW);
      digitalWrite(statusLEDPin, LOW);
      delay(3000);
    }

    // Display the timestamp and sequence on Serial Monitor
    Serial.print("Timestamp: ");
    Serial.print(timestamp);
    Serial.print(", Current Bit: ");
    Serial.println(prbs_sequence[iterationCount]);

    iterationCount++;

    if (iterationCount >= numIterations) {
```

```
      sequenceRunning = false;
      iterationCount = 0;
      digitalWrite(relayPin, LOW);
      digitalWrite(statusLEDPin, LOW);
      delay(3000);
    }
  }
}
```

SM3 Arduino script used to control the valve operation

SM4 Mean bacterial count (technical replicates) for the inoculated broth samples across three days and associated cGC peak areas for inoculated and control solutions

	Average microbial concentration and standard deviation (SD) (log CFU/mL)					
	Day 1		Day 2		Day 3	
Time points (hours)	Mean	SD	Mean	SD	Mean	SD
5	1.82	1.46	2.48	1.46	2.00	1.70
8	5.65	4.70	4.93	3.94	5.19	4.44
11	8.57	7.55	6.76	6.02	8.03	7.24
14	8.81	8.55	9.41	8.76	8.52	7.76

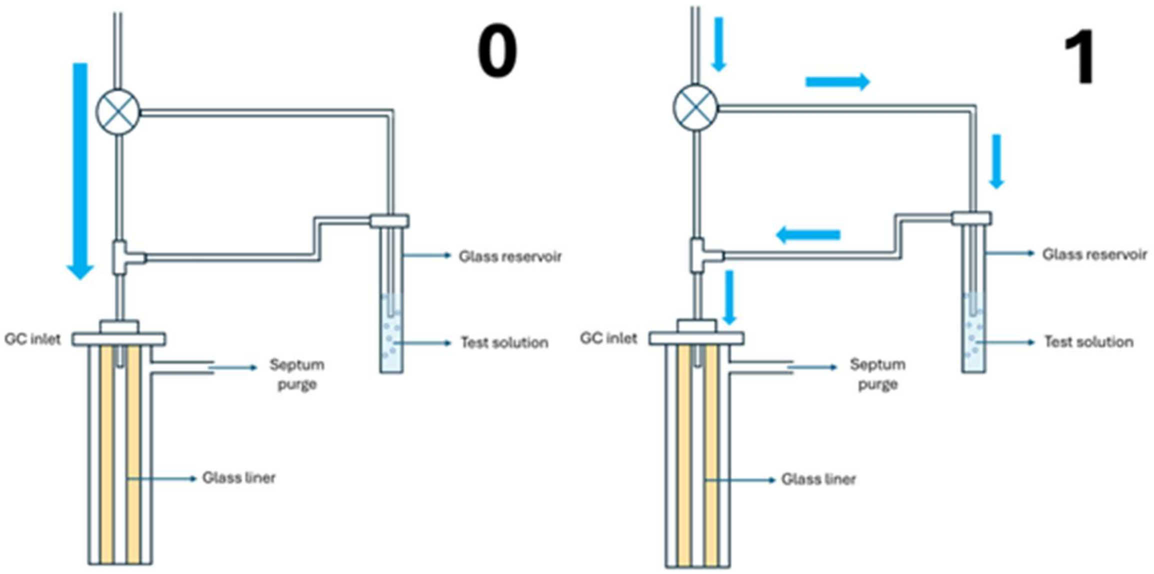

SM5 Diagram depicting the gas flow in two-states: (0) valve closed directs the gas flow to the inlet; (1) valve open directs the gas flow through the glass vial into the sample and pushing a portion of headspace to the GC inlet

a) 60 s

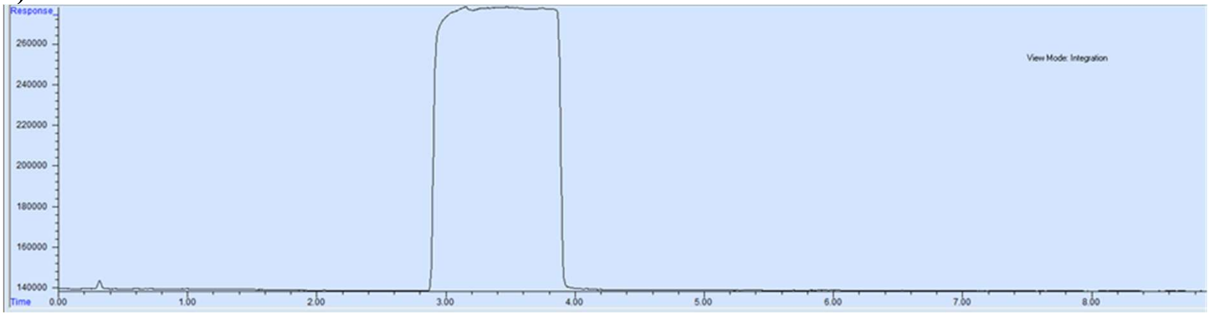

b) 30 s

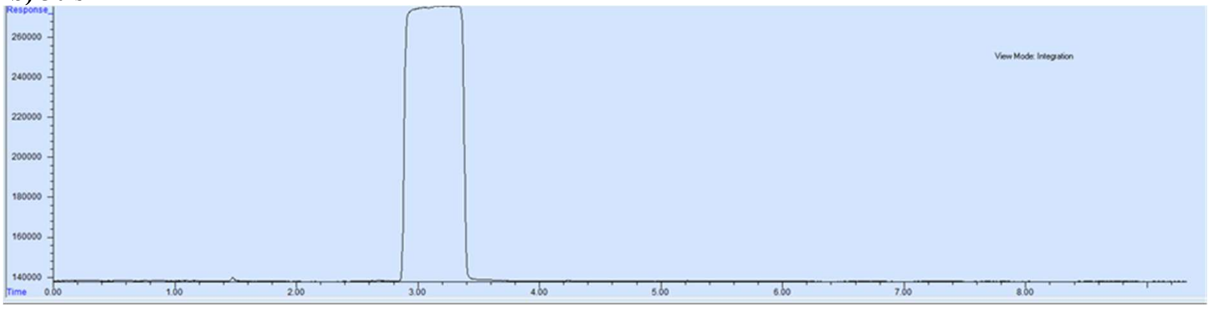

c) 10 s

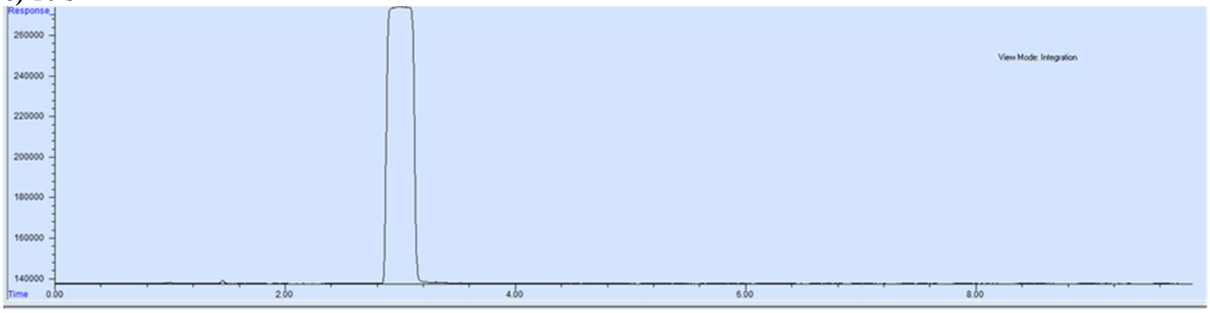

d) 3 s

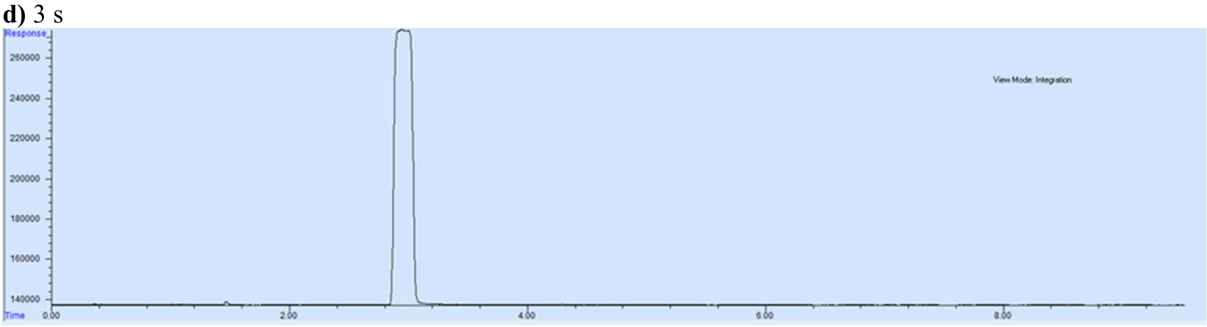

e) 600 ms

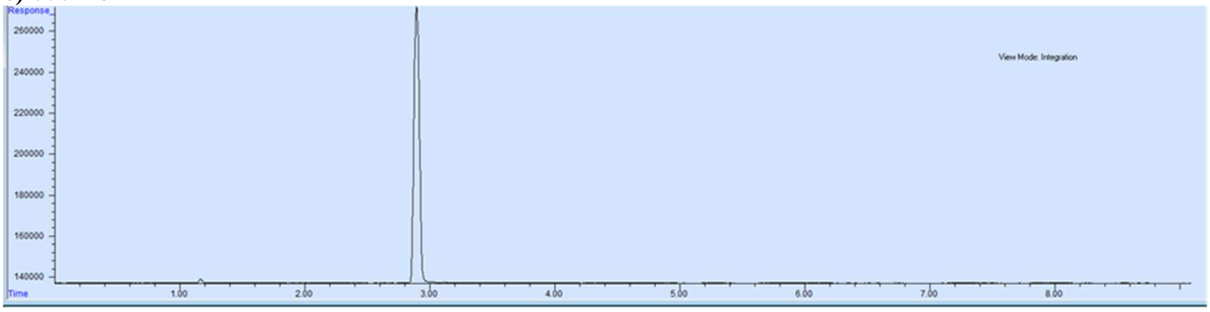

SM6 Chromatograms demonstrating the peak with various time intervals: a) 60s, b) 30 s, c) 10 s, d) 3 s, and e) 600 ms. Acetone test solution was injected. X-axis represent time in mins.

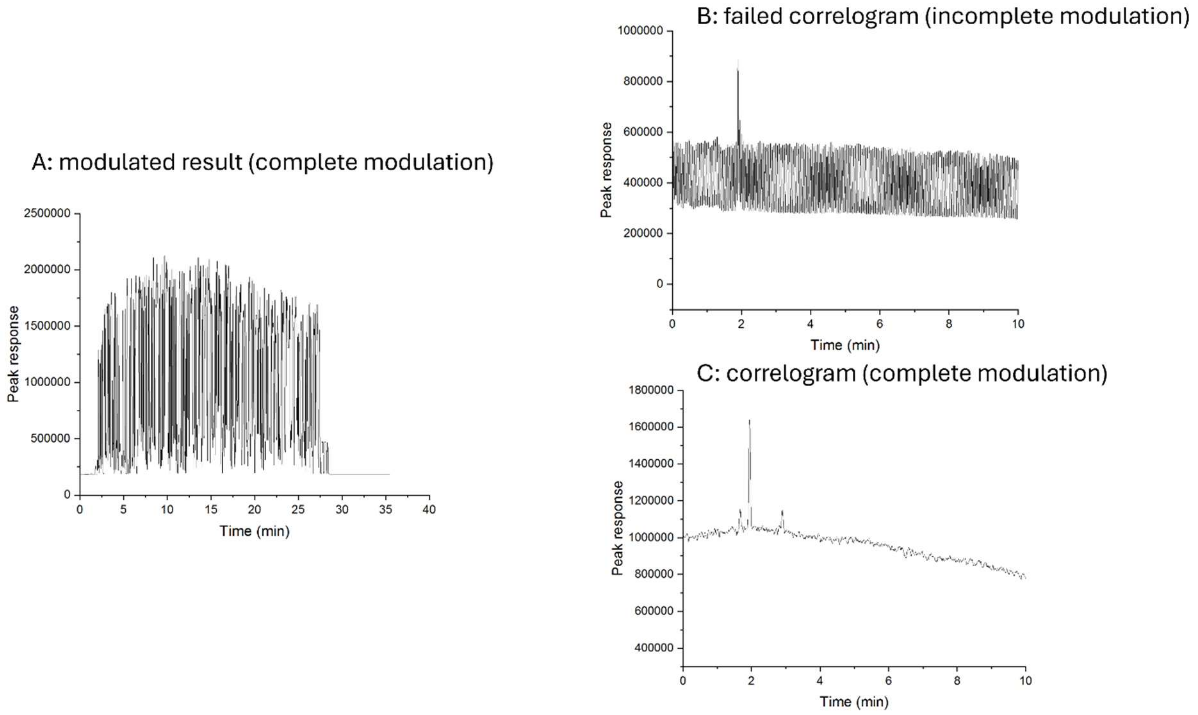

SM7 Modulated result (A) correlogram with incomplete modulation (B) and demodulated correlogram with complete modulation (C). Compounds injected acetone, ethanol, and 1-propanol.

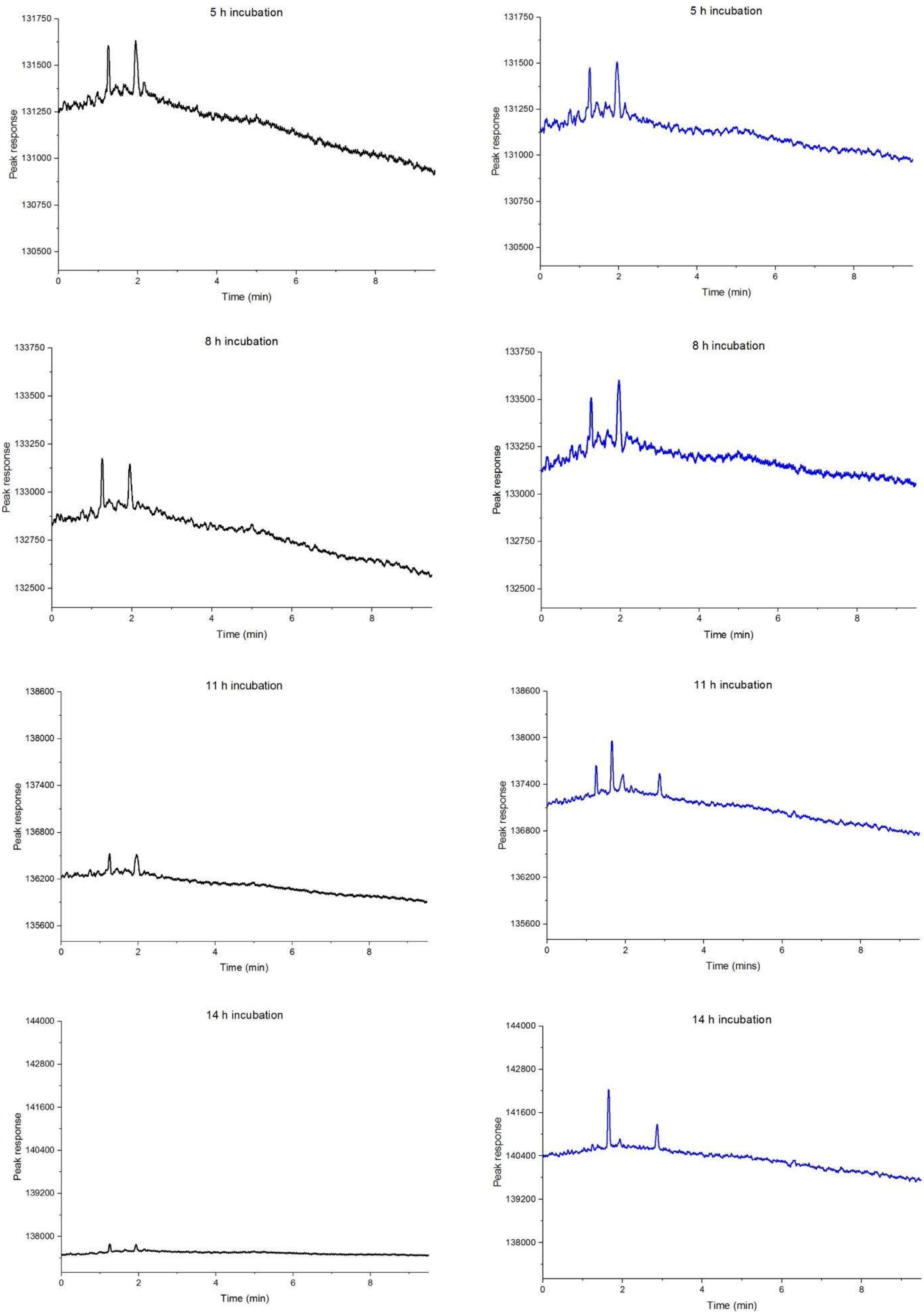

SM8 Correlograms comparing the control and the inoculated samples at all time points. Black LHS (left) result represents control samples while blue RHS (right) represents *E. coli* samples.
